# Closing the loop on the GABA shunt in plants: are GABA metabolism and signaling entwined?

**DOI:** 10.3389/fpls.2015.00419

**Published:** 2015-06-09

**Authors:** Simon Michaeli, Hillel Fromm

**Affiliations:** Department of Molecular Biology and Ecology of Plants, Faculty of Life Sciences, Tel Aviv University, Tel Aviv, Israel

**Keywords:** γ-Aminobutyric acid, GABA shunt, glutamate decarboxylase, tricarboxylic acids, *Arabidopsis*, stress

## Abstract

γ-Aminobutyric acid (GABA) is a non-proteinogenic amino acid that is found in uni- and multi-cellular organisms and is involved in many aspects of plant life cycle. GABA metabolism occurs by the action of evolutionary conserved enzymes that constitute the GABA shunt, bypassing two steps of the TCA cycle. The central position of GABA in the interface between plant carbon and nitrogen metabolism is well established. In parallel, there is evidence to support a role for GABA as a signaling molecule in plants. Here we cover some of the recent findings on GABA metabolism and signaling in plants and further suggest that the metabolic and signaling aspects of GABA may actually be inseparable.

## GABA is Associated with Primary Nitrogen and Carbon Metabolism and is Tightly Linked to the TCA Cycle

γ-Aminobutyric acid (GABA) is a four-carbon (C) non-proteinogenic amino acid (AA) that was first discovered in plants over half a century ago (Steward et al., 1949). GABA was later revealed in mammalian brain (Roberts and Frankel, 1950) and soon main interest in GABA shifted to animals when it was shown to play a major role in neurotransmission (Roberts et al., 1960). GABA has since been investigated in several organisms including bacteria, fungi, plants and animals (Bouché et al., 2003b). In both eukaryotes and bacteria GABA is a significant component of the free AA pool (Shelp et al., 1999) and the enzymes involved in GABA metabolism are conserved (Metzer and Halpern, 1990; Tillakaratne et al., 1995; Bown and Shelp, 1997; Kumar et al., 2000). Similar enzymes (e.g., glutamate decarboxylase) are found also in archaea, however their roles in GABA metabolism are questionable (Tomita et al., 2014). Interest in plant GABA increased mainly following observations of rapid elevation of its levels under abiotic stresses. Nevertheless, the roles of GABA under these conditions is not clear (Kinnersley and Turano, 2000; Fait et al., 2008). Recent combined genetics and physiological studies of the GABA shunt indicate that its function is required for proper growth in response to abiotic stresses such as low light (Michaeli et al., 2011) and salt (Renault et al., 2013).

**FIGURE 1:**
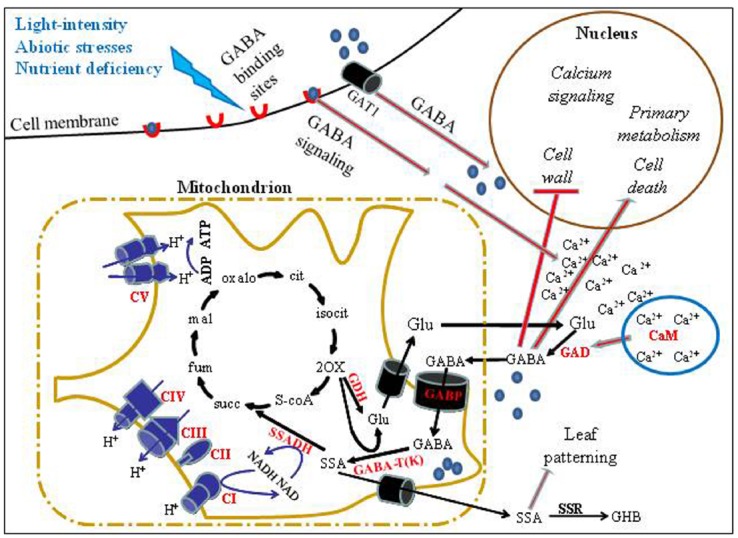
**A schematic model of GABA transport, metabolism and signaling in plant cells.** External stimuli, such as abiotic stresses and light deficiency, regulate the expression of GABA shunt-associated genes. Such stimuli may further result in increased levels of GABA, enabling its attachment to cell-surface binding sites that generate transient Ca^2+^ increase and transport into cells via high affinity GABA transporters (e.g., GAT1; Meyer et al., 2006). Consequentially, GAD may be activated via a Ca^2+^/CaM complex (Baum et al., 1993). This increase in intracellular GABA may induce the expression of several signaling and metabolism-associated genes while repressing other genes such as genes associated with cell wall-modifications. Depending on the environmental conditions, a significant proportion of cytosolic GABA may enter mitochondria through the GABA permease, AtGABP (Michaeli et al., 2011), for catabolism by GABA-T and SSADH, resulting in succinate formation to feed the TCAC and mitochondrial ETC. Alternatively, the toxic intermediate, SSA, may be transported out of the mitochondrion to form GHBA via the enzyme SSR (GHBDH). We note that recently a tonoplast Glu/Asp/GABA exchanger in tomato fruit has been reported (Snowden et al., 2015), which is not included in the presented model. Enzyme names are in bold and red, whereas the reactions they perform are indicated as black arrows. Red-filled lines indicate a regulatory effect. Blue spheres denote GABA, red crescents denote GABA receptors. Abbreviations: GDH, Glutamate dehydrogenase; succ, succinate; fum, fumarate; Mal, malate; oxalo, oxaloacetate; cit, citrate; isocit, isocitrate; 2-OX, 2-oxoglutarate (α-ketoglutarate); Glu, glutamate; CI, CII, CIII, CIV, and CV, complexes I, II, III, IV and V of the mitochondrial ETC, Respectively.

γ-Aminobutyric acid is metabolized via a short pathway known as the GABA shunt (Figure 1), which bypasses two steps of the tricarboxylic acid cycle (TCAC). GABA is mainly produced by the irreversible reaction of the cytosolic enzyme glutamate decarboxylase (GAD; EC 4.1.l.15) that consumes a proton and releases CO_2_ (Baum et al., 1993; Fait et al., 2008). However, GABA synthesis may also occur via polyamine (putrescine and spermidine) degradation (Fait et al., 2008; Shelp et al., 2012a) and possibly by a non-enzymatic reaction from proline under oxidative stress (Signorelli et al., 2015). GABA catabolism occurs in the mitochondrial matrix of multicellular organisms by the action of GABA transaminase (GABA-T; EC 2.6.1.19) to produce succinic semi-aldehyde (SSA) with the possible participation of several amino acceptors such as α-ketoglutarate (AKG), pyruvate or glyoxylate (Clark et al., 2009a; Shelp et al., 2012b). Subsequently, SSA is converted by another mitochondrial enzyme, SSA dehydrogenase (SSADH; EC 1.2.1.16) to succinate that acts both as an electron donor to the mitochondrial electron transport chain (ETC) and as a component of the TCAC (Bouché et al., 2003a; Shelp et al., 2012b). Alternatively, SSA can be converted to γ-hydroxybutyric acid (GHBA) through a GHB dehydrogenase (GHBDH) that was reported in animals and plants (Andriamampandry et al., 1998; Breitkreuz et al., 2003) and more recently in *E. coli* (Saito et al., 2009). Because AKG may serve as a precursor of glutamate and subsequently of GABA, this metabolic pathway may be viewed as bypassing two enzymatic steps of the TCAC (Figure 1), AKG dehydrogenase (AKGDH) and Succinyl Co-A ligase (SCOAL), thus termed the GABA shunt (Bown and Shelp, 1997).

Direct functional association of the GABA shunt and the TCAC was demonstrated in transgenic tomato plants with reduced activity of SCOAL (Studart-Guimaraes et al., 2007) and in potato slices treated with a specific chemical inhibitor of AKGDH (Araújo et al., 2008). Both enzymes are bypassed by the GABA shunt and in both cases elevated flux through the GABA shunt compensated for the lost activity of these TCAC enzymes. Reciprocally, mutants of the *Arabidopsis* GABP (mitochondrial GABA transporter) resulted in reduced uptake of GABA into mitochondria and increased TCAC activity (Michaeli et al., 2011). Much data accumulated regarding plant TCAC in illuminated leaves (Tcherkez et al., 2009; Sweetlove et al., 2010; Araújo et al., 2011a) where it seems to “lose” its classical cyclic metabolic form. Evidence suggests that citrate, stored from the TCAC activity of the previous night, is mainly shuttled for N assimilation (Tcherkez et al., 2009; Sweetlove et al., 2010). Interestingly, the GABA shunt, as one of the bridges between N and C metabolism, is significantly active in illuminated *Xanthium strumarium* (cocklebur) leaves as demonstrated by the high metabolic flux through it. On the other hand, a metabolic flux from AKG to succinate through the TCAC was almost undetectable (Tcherkez et al., 2009; Figure 1). In other words, the GABA shunt is apparently the major source for succinate in leaves at day time, contrary to the night-type heterotrophic plant metabolism where TCAC functions in its classical cyclic form (Sweetlove et al., 2010).

An interesting transcriptional co-response was demonstrated with *Arabidopsis* genes that encode the GABA shunt pathway (*GAD2* and *SSADH*) and other primary C metabolism associated genes, providing further support for the association of GABA with primary C metabolism in plants at both the metabolome and transcriptome levels (Fait et al., 2008). It should be noted that the genes encoding the mitochondrial GABA permease, AtGABP and the GABA catabolic enzyme SSADH are also highly co-expressed in response to stress (Michaeli et al., 2011). Changes in the cellular distribution and quantity of GABA were shown also to affect levels of several AAs in different plant species (Baum et al., 1996; Michaeli et al., 2011; Deleu et al., 2013; Shimajiri et al., 2013a; Batushansky et al., 2014; Snowden et al., 2015) and even in seeds (Fait et al., 2011). As a molecule synthesized mainly from glutamate, yet intimately associated with the TCAC, GABA is continuously suggested to be an important component in the balance between C and nitrogen (C:N) pools of plant cells (Fait et al., 2008; Renault, 2013; Batushansky et al., 2014).

## Effects of GABA on Plant Development, Organ Patterning, Interaction with Pathogens and Fertility

A signaling role for GABA in plants is continuously suggested and discussed (Bouché et al., 2003b; Bouché and Fromm, 2004; Häusler et al., 2014) and was especially highlighted following the discovery of the interesting role of GABA in pollen tube growth and guidance (Palanivelu et al., 2003) and by identifying genes that encode putative receptors that possess domains, which are structurally homologous to mammalian GABA_B_ receptors (Lacombe et al., 2001). Also, the role of GABA in regulating expression of several genes was reported: *ACC-synthase* (Kathiresan et al., 1997), *arginine-decarboxylase* (Turano et al., 1997), *14-3-3* (Lancien and Roberts, 2006), and genes encoding secreted and cell wall associated proteins (Renault et al., 2011). Consistent with a role in *ACC-Synthase* regulation, exogenously applied GABA was shown to induce ethylene emission in *Stellaria longipes* and sunflower (Kathiresan et al., 1997, 1998). Nevertheless, in our opinion, the most compelling evidence suggesting that GABA is a signaling molecule in plants is the detection of Quantum dot (QD)-mediated GABA binding sites on the surface of plant pollen and somatic protoplasts, implying the existence of GABA receptors. Importantly, the binding of the QD-GABA probes caused transient elevation in intracellular Ca^2+^ levels (Yu et al., 2006; Figure 1), which is known to induce signaling pathways in plants (Galon et al., 2010; Batistič and Kudla, 2012; Fromm and Finkler, 2015). Notably, it was recently shown that this increase in Ca^2+^ levels is the result of the modulation of Ca^2+^-permeable channels to enable proper pollen tube growth, further supporting the suggestion that the requirement for a GABA gradient along pollen tube is related to its signaling role rather than to a metabolic one (Yu et al., 2014).

Furthermore, a role for GABA as a signal between plants and bacteria is already well established (Shelp et al., 2006). GABA that accumulates in wounded plant tissues restricts quorum sensing of *Agrobacterium tumefaciens* by regulating expression of the bacterial *attKLM* operon (Chevrot et al., 2006). It was shown that plant GAD is responsible for the accumulation of the GABA signal that is transported into the bacteria via the bacterial Bra ABC transporter. Interestingly, plants with an ectopically functional GAD, which lacks the Ca^2+^/Calmodulin (Ca^2+^/CaM) binding domain, exhibited increased resistance to *Agrobacterium* infection (Chevrot et al., 2006). Increased GABA shunt activity was more recently also associated with tomato resistance to *Botrytis cinerea* (Seifi et al., 2013). In pepper (*Capsicum annuum*), the *Xanthomonas campestris* pv. *vesicatoria* effector, AvrBsT, induces hypersensitive cell death. Investigation of this effector revealed that it interacts with a pepper arginine decarboxylase (CaADC1) and their joint expression is accompanied by polyamine, nitric oxide and hydrogen peroxide bursts. Treatment of pepper with GABA significantly reduced growth of avirulent *Xanthomonas campestris*, suggesting a role for GABA in cell death promotion (Kim et al., 2013). This is consistent with an older report exhibiting GABA as a modulator of soybean (*Glycine max*) arginine decarboxylase, ultimately affecting polyamine biosynthesis (Turano et al., 1997). The association of GABA and cell death was also demonstrated in *Arabidopsis* seeds expressing a constitutively active GAD where up-regulation of cell-death-associated genes was detected. Among the over-represented categories of genes expressed in the hyperaccumulating GABA seeds were those involved in Ca^2+^-mediated signaling, redox and cysteine proteases (Fait et al., 2011). The over representation of Ca^2+^-signaling associated genes is intriguing in light of the transient change in Ca^2+^ levels caused by applying GABA to protoplasts (Yu et al., 2006). Furthermore, mutation in GABA-T (which results in GABA accumulation) was shown also to suppress plant response to E-2-Hexenal, a herbivore and pathogen associated C_6_-volatile. This volatile was shown to increase GABA levels (Mirabella et al., 2007).

γ-Aminobutyric acid accumulation also results in the down regulation of genes associated with cell-wall modifications (Renault et al., 2011; Batushansky et al., 2014). Cell wall modification are correlated with developmental changes which are exerted on plants by environmental constraints (Roppolo and Geldner, 2012). Indeed, the association of increased GABA levels with altered plant development was demonstrated extensively. Ectopically active GAD (lacking its Ca^2+^/CaM regulated autoinhibitory domain) in transgenic tobacco results in developmental abnormalities that include shortened and more branched plants. Young developing leaves exhibited a delay in greening and were narrower than normal and the plants lacked pollen. Histological analysis revealed shorter cells in the stem cortex parenchyma of these lines, which could in part explain the short plant phenotype (Baum et al., 1996). These observations are consistent with more recent evidence from GABA-T mutants displaying cell elongation defects and dwarfism in *Arabidopsis* and tomato (Renault et al., 2011; Koike et al., 2013), especially under salt stress (Renault et al., 2013). Immuno-localization of GABA in pine seedlings suggests a role for GABA in vascular development (Molina-Rueda et al., 2015). Moreover, *Arabidopsis ssadh* mutants are significantly dwarfed, a phenotype that can be rescued by a second mutation in *GABA-T*, which encodes the enzyme that functions upstream of SSADH in the mitochondrial GABA catabolism process (Ludewig et al., 2008). Interestingly, SSADH was implicated in *Arabidopsis* patterning along the abaxial/adaxial axis and these patterning defects were recovered in an *ssadh*/*gaba-t* double mutant (Toyokura et al., 2011). These authors suggested that it is the SSA intermediate between GABA and succinate that is mediating this patterning process (Toyokura et al., 2011, 2012).

In conclusion, GABA levels can readily be affected by activating plant GAD (which is tightly regulated by Ca^2+^/CaM), by suppressing its catabolism (through suppression of GABA-T), by exogenous application of GABA or by interrupting the cellular C:N balance (through suppressing of primary metabolism related enzymes such as TCAC enzymes). Such changes in GABA levels result in plant responses at the metabolic, transcriptional and developmental levels, which are difficult to explain solely within a metabolic context. Although there is yet no direct evidence for a signaling role of GABA in plants, it’s impossible to ignore the numerous evidences that imply the existence of such signaling pathways.

## Coupling of GABA Metabolism and Signaling Under Nutrient Starvation

In order to obtain further insight into natural modulation of GABA levels, we discuss the current knowledge regarding GABA metabolism under plant starvation in comparison to metabolism in starved animals. We suggest that the metabolic and signaling functions of GABA evolved to be functionally entwined.

Metabolic profiling of *Arabidopsis* mutants impaired in enzymes that are essential for respiration under extended dark-induced C starvation revealed an increase in the content of several AAs associated with starvation-induced protein degradation (Araújo et al., 2010). On the other hand, the levels of GABA and of the TCAC intermediate, succinate, increased significantly in these mutants although both metabolites are not the product of protein degradation. The authors suggested that an increase in GABA shunt activity produces mitochondrial succinate to maintain respiration in these mutants (Araújo et al., 2010). This was also apparent in a different study that employed metabolic and transcriptional analyses to decipher mechanisms mediating the *Arabidopsis* response to dark-induced starvation. GABA was once again highlighted as the major non-proteinogenic AA with a tight correlation to succinate levels (Caldana et al., 2011). Notably, at the transcriptional level, starving plants exhibited significant reduced expression of TCAC-associated *SCOAS* genes in parallel to a significant increase in *SSADH* expression, implying an induction of the GABA shunt bypass under these circumstances (Caldana et al., 2011). Though succinate accumulation in starved plants may be explained by its use as an electron donor in the respiratory chain, the accumulation of GABA, if not merely a metabolic side effect, awaits explanation. One possibility is that the accumulation of GABA is a prerequisite for its signaling function. Let’s look at an example for such a metabo–signaling coupling of GABA under low energy status in an organism with a well-established signaling role of GABA.

In mice, the brain is responsible for inducing a hunger sensation under low (or no) food availability that will result in a behavioral response of seeking and consuming food (Atasoy et al., 2012; Wu et al., 2012). An interesting report demonstrates how specific neurons (AgRP neurons of the arcuate nucleus) modulate feeding behavior in mice by providing GABAergic input into mice brainstem (the parabrachial nucleus; PBN) and how inactivation of GABA biosynthesis (by inactivating murine Gad1) in the arcuate nucleus, or block of GABA(A) receptors in the PBN, promotes mice anorexia (Wu et al., 2009). This elegantly shows how GABA neurotransmission participates in the maintenance of energy homeostasis of the whole organism (feeding behavior) and how it is directly affected by GABA biosynthesis through the action of Gad1 (Dietrich and Horvath, 2009; Wu et al., 2009).

As autotrophic organisms, the two main aspects of plant “feeding” are photosynthesis and nutrient uptake by the root. GABA is one of few metabolites whose metabolic path is divided between the cytosol and the mitochondrion matrix. The possibility to target mitochondrial metabolism as a means to enhance photosynthesis was already discussed (Nunes-Nesi et al., 2011; Araújo et al., 2014) and shown (Araújo et al., 2011b). Notably, *in silico* analysis of optimal photosynthesis in cyanobacteria highlighted the importance of the GABA shunt, combined with an incomplete TCAC during autotrophic metabolism (Nogales et al., 2012). Remarkably, some GABA-T isoforms of rice and tomato were shown to localize in plastids (Clark et al., 2009b; Shimajiri et al., 2013b). This suggests a role for GABA or its derivatives in plastid-associated functions. Indeed exogenously applied GABA resulted in increased photosynthesis parameters is muskmelon seedlings, mainly under hypoxia stress (Xia et al., 2011).

Nevertheless, in order to more accurately compare animal feeding with plant “feeding” it is more prudent to refer also to nutrient uptake by plant roots. Two reports suggest a signaling role for GABA in nitrate uptake in *Brassica napus* roots (Beuve et al., 2004) and more recently in *Arabidopsis thaliana* (Barbosa et al., 2011). An earlier report described how GABA enhances significantly the growth of *Lemna minor* plants by increasing mineral consumption. In contrast, isomers of GABA such as 3-aminobutyric acid and 2-aminobutyric acid inhibited plant growth. Remarkably, GABA mediated promotion of *Lemna* growth was inhibited by bicuculline and picrotoxin, which are competitive and non-competitive antagonists of GABA receptors in the mammalian central nerve system, respectively. Consistent with these findings, Baclofen, a known GABA agonist in animals, significantly increased GABA mediated promotion of *Lemna* growth suggesting the existence of GABA receptors in plants that participate in nutrient uptake and eventually affect plant growth (Kinnersley and Lin, 2000). Moreover, a role for GABA in the up-regulation of nodule activity was also suggested (Sulieman and Schulze, 2010) followed by evidences for a role of GABA in increasing the efficiency of symbiotic N_2_ fixation in legumes (Sulieman, 2011). Thus, it seems that GABA levels increase during plant starvation and energetically demanding stresses. Importantly, GABA seems to induce plant responses that may aid in replenishing the energetic supply, very similar to the action of GABA in mammals where it modulates feeding behavior. The already mentioned effect of GABA on exerting cell-elongation arrest may be important in parallel in order to save cellular energy. Thus, the combined GABA associated effects of modulating photosynthesis and nutrient uptake, in parallel to growth arrest, may be pivotal to ensure plant survival under energetically demanding stresses.

## Concluding Remarks

γ-Aminobutyric acid is a major metabolic component in the interface between C and N metabolism. As such, its levels are “sensitive” to the availability of both essential elements, which makes it an excellent sensor for the energetic state of the cell. Thus, GABA seems ideal to serve as a signal that participates in a pathway that “instructs” the organism whether to try and gain more energy, or whether to initiate processes to cope with excess energy. Also, it seems that GABA is a major alternative pathway to which C skeleton is directed depending on the environment, growth stage, and tissue specificity. These reports summarized here suggest that from an evolutionary perspective, GABA metabolism predated signaling, and that mechanistically, GABA metabolism underlies its signaling functions. This is consistent with a work that integrated metabolomics with transcript and enzyme activity profiling of plants undergoing diurnal cycles. The authors concluded that “…correlation between metabolites and transcripts are due to regulation of gene expression by metabolites, rather than metabolites being changed as a consequence of a change in gene expression” (Gibon et al., 2006). Future research should shed light on the manner by which physiological increase in GABA levels, either by GAD induction, repression of catabolism or increased flux in the direction of the GABA shunt, affect plant resilience and development. Identification of the cell-surface localized GABA receptors, as well as identifying components that participate in GABA-mediated gene control is essential to this end.

### Conflict of Interest Statement

The authors declare that the research was conducted in the absence of any commercial or financial relationships that could be construed as a potential conflict of interest.

## References

[B1] Andriamampandry C. Siffert J. C. Schmitt M. Garnier J. M. Staub A. Muller C. (1998). Cloning of a rat brain succinic semialdehyde reductase involved in the synthesis of the neuromodulator γ-hydroxybutyrate. Biochem. J. 334, 43–50. 9693100 10.1042/bj3340043 PMC1219659

[B2] Araújo W. L. Ishizaki K. Nunes-Nesi A. Larson T. R. Tohge T. Krahnert I. (2010). Identification of the 2-hydroxyglutarate and isovaleryl-CoA dehydrogenases as alternative electron donors linking lysine catabolism to the electron transport chain of *Arabidopsis* mitochondria. Plant Cell 22, 1549–1563. 10.1105/tpc.110.075630 20501910 PMC2899879

[B3] Araújo W. L. Nunes-Nesi A. Fernie A. (2014). On the role of plant mitochondrial metabolism and its impact on photosynthesis in both optimal and sub-optimal growth conditions. Photosyn. Res. 119, 141–156. 10.1007/s11120-013-9807-4 23456269

[B4] Araújo W. L. Nunes-Nesi A. Nikoloski Z. Sweetlove L. J. Fernie A. R. (2011a). Metabolic control and regulation of the tricarboxylic acid cycle in photosynthetic and heterotrophic plant tissues. Plant Cell Environ. 35, 1–21. 10.1111/j.1365-3040.2011.02332.x 21477125

[B5] Araújo W. L. Nunes-Nesi A. Osorio S. Usadel B. R. Fuentes D. Nagy R. K. (2011b). Antisense inhibition of the iron-sulphur subunit of succinate dehydrogenase enhances photosynthesis and growth in tomato via an organic acid-mediated effect on stomatal aperture. Plant Cell 23, 600–627. 10.1105/tpc.110.081224 21307286 PMC3077794

[B6] Araújo W. L. Nunes-Nesi A. Trenkamp S. Bunik V. I. Fernie A. R. (2008). Inhibition of 2-oxoglutarate dehydrogenase in potato tuber suggests the enzyme is limiting for respiration and confirms its importance in nitrogen assimilation. Plant Physiol. 148, 1782–1796. 10.1104/pp.108.126219 18842826 PMC2593666

[B7] Atasoy D. Betley J. N. Su H. H. Sternson S. M. (2012). Deconstruction of a neural circuit for hunger. Nature 488, 172–177. 10.1038/nature11270 22801496 PMC3416931

[B8] Barbosa J. M. Singh N. K. Cherry J. H. Locy R. D. (2011). Nitrate uptake and utilization is modulated by exogenous γ-aminobutyric acid in *Arabidopsis thaliana* seedlings. Plant Physiol. Biochem. 48, 443–450. 10.1016/j.plaphy.2010.01.020 20303774

[B9] Batistič O. Kudla J. (2012). Analysis of calcium signaling pathways in plants. Biochim. Biophys. Acta 1820, 1283–1293. 10.1016/j.bbagen.2011.10.012 22061997

[B10] Batushansky A. Kirma M. Grillich N. Toubiana D. Pham P. A. Balbo I. (2014). Combined transcriptomics and metabolomics of *Arabidopsis thaliana* seedlings exposed to exogenous GABA suggest its role in plants is predominantly metabolic. Mol. Plant 7, 1065–1068. 10.1093/mp/ssu017 24553152

[B11] Baum G. Chen Y. Arazi T. Takatsuji H. Fromm H. (1993). A plant glutamate decarboxylase containing a calmodulin binding domain. Cloning, sequence, and functional analysis. J. Biol. Chem. 268, 19610–19617 8366104

[B12] Baum G. Lev-Yadun S. Fridmann Y. Arazi T. Katsnelson H. Zik M. (1996). Calmodulin binding to glutamate decarboxylase is required for regulation of glutamate and GABA metabolism and normal development in plants. EMBO J. 15, 2988–2996 8670800 PMC450240

[B13] Beuve N. Rispail N. Laine P. Cliquet J. B. Ourry A. Le Deunff E. (2004). Putative role of γ-aminobutyric acid (GABA) as a long-distance signal in up-regulation of nitrate uptake in *Brassica napus* L. Plant Cell Environ. 27, 1035–1046. 10.1111/j.1365-3040.2004.01208.x

[B14] Bouché N. Fait A. Bouchez D. Moller S. G. Fromm H. (2003a). Mitochondrial succinic-semialdehyde dehydrogenase of the γ-aminobutyrate shunt is required to restrict levels of reactive oxygen intermediates in plants. Proc. Natl. Acad. Sci. U.S.A. 100, 6843–6848. 10.1073/pnas.1037532100 12740438 PMC164534

[B15] Bouché N. Lacombe B. Fromm H. (2003b). GABA signaling: a conserved and ubiquitous mechanism. Trends Cell Biol. 13, 607–610. 10.1016/j.tcb.2003.10.001 14624837

[B16] Bouché N. Fromm H. (2004). GABA in plants: just a metabolite? Trends Plant Sci. 9, 110–115. 10.1016/j.tplants.2004.01.006 15003233

[B17] Bown A. W. Shelp B. J. (1997). The metabolism and functions of γ-aminobutyric acid. Plant Physiol. 115, 1–5. 12223787 10.1104/pp.115.1.1 PMC158453

[B18] Breitkreuz K. E. Allan W. L. Van Cauwenberghe O. R. Jakobs C. Talibi D. Andre B. (2003). A novel γ-hydroxybutyrate dehydrogenase. J. Biol. Chem. 278, 41552–41556. 10.1074/jbc.M305717200 12882961

[B19] Caldana C. Degenkolbe T. Cuadros-Inostroza A. Klie S. Sulpice R. Leisse A. (2011). High-density kinetic analysis of the metabolomic and transcriptomic response of *Arabidopsis* to eight environmental conditions. Plant J. 67, 869–884. 10.1111/j.1365-313X.2011.04640.x 21575090

[B20] Chevrot R. Rosen R. Haudecoeur E. Cirou A. Shelp B. J. Ron E. (2006). GABA controls the level of quorum-sensing signal in *Agrobacterium tumefaciens*. Proc. Natl. Acad. Sci. U.S.A. 103, 7460–7464. 10.1073/pnas.0600313103 16645034 PMC1464361

[B21] Clark S. M. Di Leo R. Dhanoa P. K. Van Cauwenberghe O. R. Mullen R. T. Shelp B. J. (2009a). Biochemical characterization, mitochondrial localization, expression, and potential functions for an *Arabidopsis* γ-aminobutyrate transaminase that utilizes both pyruvate and glyoxylate. J. Exp. Bot. 60, 1743–1757. 10.1093/jxb/erp044 19264755 PMC2671622

[B22] Clark S. M. Di Leo R. Van Cauwenberghe O. R. Mullen R. T. Shelp B. J. (2009b). Subcellular localization and expression of multiple tomato γ-aminobutyrate transaminases that utilize both pyruvate and glyoxylate. J. Exp. Bot. 60, 3255–3267. 10.1093/jxb/erp161 19470656 PMC2718222

[B23] Deleu C. Faes P. Niogret M.-F. O. Bouchereau A. (2013). Effects of the inhibitor of the γ-aminobutyrate-transaminase, vinyl-γ-aminobutyrate, on development and nitrogen metabolism in *Brassica napus* seedlings. Plant Physiol. Biochem. 64, 60–69. 10.1016/j.plaphy.2012.12.007 23370302

[B24] Dietrich M. O. Horvath T. L. (2009). GABA keeps up an appetite for life. Cell 137, 1177–1179. 10.1016/j.cell.2009.06.002 19563747

[B25] Fait A. Fromm H. Walter D. Galili G. Fernie A. R. (2008). Highway or byway: the metabolic role of the GABA shunt in plants. Trends Plant Sci. 13, 14–19. 10.1016/j.tplants.2007.10.005 18155636

[B26] Fait A. Nesi A. N. Angelovici R. Lehmann M. Pham P. A. Song L. (2011). Targeted enhancement of glutamate-to-γ-aminobutyrate conversion in *Arabidopsis* seeds affects carbon-nitrogen balance and storage reserves in a development-dependent manner. Plant Physiol. 157, 1026–1042. 10.1104/pp.111.179986 21921115 PMC3252140

[B27] Fromm H. Finkler A. (2015). Repression and de-repression of gene expression in the plant immune response: the complexity of modulation by Ca^2+^ and calmodulin. Mol. Plant 8, 671–673. 10.1016/j.molp.2015.01.019 25633528

[B28] Galon Y. Finkler A. Fromm H. (2010). Calcium-regulated transcription in plants. Mol. Plant 3, 653–669. 10.1093/mp/ssq019 20457642

[B29] Gibon Y. Usadel B. Blaesing O. Kamlage B. Hoehne M. Trethewey R. (2006). Integration of metabolite with transcript and enzyme activity profiling during diurnal cycles in *Arabidopsis* rosettes. Genome Biol. 7, 1–23. 10.1186/gb-2006-7-8-r76 16916443 PMC1779593

[B30] Häusler R. E. Ludewig F. Krueger S. (2014). Amino acids–a life between metabolism and signaling. Plant Sci. 229, 225–237. 10.1016/j.plantsci.2014.09.011 25443849

[B31] Kathiresan A. Miranda J. Chinnappa C. C. Reid D. M. (1998). γ-aminobutyric acid promotes stem elongation in *Stellaria longipes*: the role of ethylene. Plant Growth Regul. 26, 131–137. 10.1023/A:1006107815064

[B32] Kathiresan A. Tung P. Chinnappa C. C. Reid D. M. (1997). γ-aminobutyric acid stimulates ethylene biosynthesis in sunflower. Plant Physiol. 115, 129–135. 9306696 10.1104/pp.115.1.129 PMC158468

[B33] Kim N. H. Kim B. S. Hwang B. K. (2013). Pepper arginine decarboxylase is required for polyamine and γ-aminobutyric acid signaling in cell death and defense response. Plant Physiol. 162, 2067–2083. 10.1104/pp.113.217372 23784462 PMC3729783

[B34] Kinnersley A. M. Lin F. (2000). Receptor modifiers indicate that 4-aminobutyric acid (GABA) is a potential modulator of ion transport in plants. Plant Growth Regul. 32, 65–76. 10.1023/a:1006305120202

[B35] Kinnersley A. M. Turano F. J. (2000). γ aminobutyric acid (GABA) and plant responses to stress. Crit. Rev. Plant Sci. 19, 479–509. 10.1080/07352680091139277

[B36] Koike S. Matsukura C. Takayama M. Asamizu E. Ezura H. (2013). Suppression of γ-aminobutyric acid (GABA) transaminases induces prominent GABA accumulation, dwarfism and infertility in the tomato (*Solanum lycopersicum* L.). Plant Cell Physiol. 54, 793–807. 10.1093/pcp/pct035 23435575

[B37] Kumar S. Punekar N. S. Satyanarayan V. Venkatesh K. V. (2000). Metabolic fate of glutamate and evaluation of flux through the 4-aminobutyrate (GABA) shunt in *Aspergillus niger*. Biotechnol. Bioeng. 67, 575–584. 10.1002/(SICI)1097-0290(20000305)67:5<575::AID-BIT8>3.0.CO;2-L 10649232

[B38] Lacombe B. Becker D. Hedrich R. Desalle R. Hollmann M. Kwak J. M. (2001). The identity of plant glutamate receptors. Science 292, 1486–1487. 10.1126/science.292.5521.1486b 11379626

[B39] Lancien M. Roberts M. R. (2006). Regulation of *Arabidopsis thaliana* 14-3-3 gene expression by γ-aminobutyric acid. Plant Cell Environ. 29, 1430–1436. 10.1111/j.1365-3040.2006.01526.x 17080964

[B40] Ludewig F. Hüser A. Fromm H. Beauclair L. Bouché N. (2008). Mutants of GABA transaminase (POP2) suppress the severe phenotype of succinic semialdehyde dehydrogenase (ssadh) Mutants in *Arabidopsis*. PLoS ONE 3:e3383. 10.1371/journal.pone.0003383 18846220 PMC2557145

[B41] Metzer E. Halpern Y. S. (1990). *In vivo* cloning and characterization of the gabCTDP gene cluster of *Escherichia coli* K-12. J. Bacteriol. 172, 3250–3256. 2188954 10.1128/jb.172.6.3250-3256.1990 PMC209132

[B42] Meyer A. Eskandari S. Grallath S. Rentsch D. (2006). AtGAT1, a high affinity transporter for γ-aminobutyric acid in *Arabidopsis thaliana*. J. Biol. Chem. 281, 7197–7204. 10.1074/jbc.M510766200 16407306 PMC3009663

[B43] Michaeli S. Fait A. Lagor K. Nunes-Nesi A. Grillich N. Yellin A. (2011). A mitochondrial GABA permease connects the GABA shunt and the TCA cycle, and is essential for normal carbon metabolism. Plant J. 67, 485–498. 10.1111/j.1365-313X.2011.04612.x 21501262

[B44] Mirabella R. Rauwerda H. Struys E. A. Jakobs C. Triantaphylides C. Haring M. A. (2007). The *Arabidopsis* her1 mutant implicates GABA in E-2-hexenal responsiveness. Plant J. 53, 197–213. 10.1111/j.1365-313X.2007.03323.x 17971036

[B45] Molina-Rueda J. S. Pascual M. N. Pissarra J. Gallardo F. (2015). A putative role for γ-aminobutyric acid (GABA) in vascular development in pine seedlings. Planta 241, 257–267. 10.1007/s00425-014-2157-4 25183257

[B46] Nogales J. Gudmundsson S. Knight E. M. Palsson B. O. Thiele I. (2012). Detailing the optimality of photosynthesis in cyanobacteria through systems biology analysis. Proc. Natl. Acad. Sci. U.S.A. 109, 2678–2683. 10.1073/pnas.1117907109 22308420 PMC3289291

[B47] Nunes-Nesi A. Araújo W. L. Fernie A. R. (2011). Targeting mitochondrial metabolism and machinery as a means to enhance photosynthesis. Plant Physiol. 155, 101–107. 10.1104/pp.110.163816 20966153 PMC3075771

[B48] Palanivelu R. Brass L. Edlund A. F. Preuss D. (2003). Pollen tube growth and guidance is regulated by POP2, an *Arabidopsis* gene that controls GABA levels. Cell 114, 47–59. 10.1016/S0092-8674(03)00479-3 12859897

[B49] Renault H. (2013). Fiat lux! Phylogeny and bioinformatics shed light on GABA functions in plants. Plant Signal. Behav. 8, e24274. 10.4161/psb.24274 23518583 PMC3909035

[B50] Renault H. El Amrani A. Berger A. Mouille G. Soubigou-Taconnat L. Bouchereau A. (2013). γ-aminobutyric acid transaminase deficiency impairs central carbon metabolism and leads to cell wall defects during salt stress in *Arabidopsis* roots. Plant Cell Environ. 36, 1009–1018. 10.1111/pce.12033 23148892

[B51] Renault H. El Amrani A. Palanivelu R. Updegraff E. P. Yu A. S. Renou J.-P. (2011). GABA accumulation causes cell elongation defects and a decrease in expression of genes encoding secreted and cell wall-related proteins in *Arabidopsis thaliana*. Plant Cell Physiol. 52, 894–908. 10.1093/pcp/pcr041 21471118 PMC3093128

[B52] Roberts E. Eidelberg E. Carl C. P. John R. S. (1960). Metabolic and neurophysiological roles of γ-aminobutyric acid. Int. Rev. Neurobiol. 2, 279–332. 13742140 10.1016/s0074-7742(08)60125-7

[B53] Roberts E. Frankel S. (1950). γ-aminobutyric acid in brain: its formation from glutamic-acid. J. Biol. Chem. 187, 55–63. 14794689

[B54] Roppolo D. Geldner N. (2012). Membrane and walls: who is master, who is servant? Curr. Opin. Plant Biol. 15, 608–617. 10.1016/j.pbi.2012.09.009 23026117

[B55] Saito N. Robert M. Kochi H. Matsuo G. Kakazu Y. Soga T. (2009). Metabolite profiling reveals YihU as a novel hydroxybutyrate dehydrogenase for alternative succinic semialdehyde metabolism in *Escherichia coli*. J. Biol. Chem. 284, 16442–16451. 10.1074/jbc.M109.002089 19372223 PMC2713508

[B56] Seifi H. S. Curvers K. De Vleesschauwer D. Delaere I. Aziz A. Höfte M. (2013). Concurrent overactivation of the cytosolic glutamine synthetase and the GABA shunt in the ABA-deficient sitiens mutant of tomato leads to resistance against *Botrytis cinerea*. New Phytol. 199, 490–504. 10.1111/nph.12283 23627463

[B57] Shelp B. J. Bown A. W. Faure D. (2006). Extracellular γ-aminobutyrate mediates communication between plants and other organisms. Plant Physiol. 142, 1350–1352. 10.1104/pp.106.088955 17151138 PMC1676054

[B58] Shelp B. J. Bown A. W. Mclean M. D. (1999). Metabolism and functions of γ-aminobutyric acid. Trends Plant Sci. 4, 446–452. 10.1016/S1360-1385(99)01486-7 10529826

[B59] Shelp B. J. Bozzo G. G. Trobacher C. P. Zarei A. Deyman K. L. Brikis C. J. (2012a). Hypothesis/review: contribution of putrescine to 4-aminobutyrate (GABA) production in response to abiotic stress. Plant Sci. 193–194, 130–135. 10.1016/j.plantsci.2012.06.001 22794926

[B60] Shelp B. J. Mullen R. T. Waller J. C. (2012b). Compartmentation of GABA metabolism raises intriguing questions. Trends Plant Sci. 17, 57–59. 10.1016/j.tplants.2011.12.006 22226724

[B61] Shimajiri Y. Oonishi T. Ozaki K. Kainou K. Akama K. (2013a). Genetic manipulation of the γ-aminobutyric acid (GABA) shunt in rice: overexpression of truncated glutamate decarboxylase (GAD2) and knockdown of γ-aminobutyric acid transaminase (GABA-T) lead to sustained and high levels of GABA accumulation in rice kernels. Plant Biotechnol. J. 11, 594–604. 10.1111/pbi.12050 23421475

[B62] Shimajiri Y. Ozaki K. Kainou K. Akama K. (2013b). Differential subcellular localization, enzymatic properties and expression patterns of γ-aminobutyric acid transaminases (GABA-Ts) in rice (*Oryza sativa*). J. Plant Physiol. 170, 196–201. 10.1016/j.jplph.2012.09.007 23122787

[B63] Signorelli S. Dans P. D. Coitiño E. L. Borsani O. Monza J. (2015). Connecting proline and γ-aminobutyric acid in stressed plants through non-enzymatic reactions. PLoS ONE 10:e0115349. 10.1371/journal.pone.0115349 25775459 PMC4361682

[B64] Snowden C. J. Thomas B. Baxter C. J. Smith J. A. C. Sweetlove L. J. (2015). A tonoplast Glu/Asp/GABA exchanger that affects tomato fruit amino acid composition. Plant J. 81, 651–660. 10.1111/tpj.12766 25602029 PMC4950293

[B65] Steward F. C. Thompson J. F. Dent C. E. (1949). γ-aminobutyric acid: a constituent of the potato tuber? Science 110, 439–440.

[B66] Studart-Guimaraes C. Fait A. Nunes-Nesi A. Carrari F. Usadel B. Fernie A. R. (2007). Reduced expression of succinyl-coenzyme A ligase can be compensated for by up-regulation of the γ-aminobutyrate shunt in illuminated tomato leaves. Plant Physiol. 145, 626–639. 10.1104/pp.107.103101 17885090 PMC2048777

[B67] Sulieman S. (2011). Does GABA increase the efficiency of symbiotic N_2_ fixation in legumes? Plant Signal. Behav. 6, 32–36. 10.4161/psb.6.1.14318 21307661 PMC3122002

[B68] Sulieman S. Schulze J. (2010). Phloem-derived γ-aminobutyric acid (GABA) is involved in upregulating nodule N_2_ fixation efficiency in the model legume *Medicago truncatula*. Plant Cell Environ. 33, 2162–2172. 10.1111/j.1365-3040.2010.02214.x 20716066

[B69] Sweetlove L. J. Beard K. F. M. Nunes-Nesi A. Fernie A. R. Ratcliffe R. G. (2010). Not just a circle: flux modes in the plant TCA cycle. Trends Plant Sci. 15, 462–470. 10.1016/j.tplants.2010.05.006 20554469

[B70] Tcherkez G. Mahé A. Gauthier P. Mauve C. Gout E. Bligny R. (2009). In folio respiratory fluxomics revealed by 13C isotopic labeling and H/D isotope effects highlight the noncyclic nature of the tricarboxylic acid “cycle” in illuminated leaves. Plant Physiol. 151, 620–630. 10.1104/pp.109.142976 19675152 PMC2754646

[B71] Tillakaratne N. J. K. Medina-Kauwe L. Gibson K. M. (1995). γ-aminobutyric acid (GABA) metabolism in mammalian neural and nonneural tissues. Comp. Biochem. Physiol. A Physiol. 112, 247–263. 7584821 10.1016/0300-9629(95)00099-2

[B72] Tomita H. Yokooji Y. Ishibashi T. Imanaka T. Atomi H. (2014). An archaeal glutamate decarboxylase homolog functions as an aspartate decarboxylase and is involved in β-alanine and coenzyme A biosynthesis. J. Bacteriol. 196, 1222–1230. 10.1128/JB.01327-3 24415726 PMC3957723

[B73] Toyokura K. Hayashi M. Nishimura M. Okada K. (2012). Adaxial–abaxial patterning: a novel function of the GABA shunt. Plant Signal. Behav. 7, 705–707. 10.4161/psb.20346 22751326 PMC3583945

[B74] Toyokura K. Watanabe K. Oiwaka A. Kusano M. Tameshige T. Tatematsu K. (2011). Succinic semialdehyde dehydrogenase is involved in the robust patterning of *Arabidopsis* leaves along the adaxial–abaxial axis. Plant Cell Physiol. 52, 1340–1353. 10.1093/pcp/pcr079 21690177

[B75] Turano F. J. Kramer G. F. Wang C. Y. (1997). The effect of methionine, ethylene, and polyamine catabolic intermediates on polyamine accumulation in detached soybean leaves. Physiol. Plant. 101, 510–518. 10.1111/j.1399-3054.1997.tb01031.x

[B76] Wu Q. Boyle M. P. Palmiter R. D. (2009). Loss of GABAergic signaling by AgRP neurons to the parabrachial nucleus leads to starvation. Cell 137, 1225–1234. 10.1016/j.cell.2009.04.022 19563755 PMC2729323

[B77] Wu Q. Clark M. S. Palmiter R. D. (2012). Deciphering a neuronal circuit that mediates appetite. Nature 483, 594–597. 10.1038/nature10899 22419158 PMC4000532

[B78] Xia Q.-P. Gao H.-B. Li J.-R. (2011). Effects of γ-aminobutyric acid on the photosynthesis and chlorophyll fluorescence parameters of muskmelon seedlings under hypoxia stress. Chin. J. Appl. Ecol. 22, 999–1006. 21774324

[B79] Yu G.-H. Zou J. Feng J. Peng X. B. Wu J.-Y. Wu Y.-L. (2014). Exogenous γ-aminobutyric acid (GABA) affects pollen tube growth via modulating putative Ca^2+^-permeable membrane channels and is coupled to negative regulation on glutamate decarboxylase. J. Exp. Bot. 65, 3235–3248. 10.1093/jxb/eru171 24799560 PMC4071839

[B80] Yu G. Liang J. He Z. Sun M. (2006). Quantum dot-mediated detection of γ-aminobutyric acid binding sites on the surface of living pollen protoplasts in tobacco. Chem. Biol. 13, 723–731. 10.1016/j.chembiol.2006.05.007 16873020

